# Trephining for Epilepsy

**Published:** 1892-08-13

**Authors:** 


### SURGERY.

### TREPHINING FOR EPILEPSY.

No department of surgery has made more distinct progress of late years than cranial surgery. Yet, perhaps, the advance has appeared more marked than we are really entitled to claim, for progression has been rather in the direction of successfully localising the lesions than in successfully treating them. But within the last few months so many cases of traumatic epilepsy have been cured by the operation of trephining that cranial surgery may perhaps be said to have emerged from its backward state.

The whole question of the surgical treatment of epilepsy has been reviewed by Professor Sachs, of New York.* He remarks at the outset that, unfortunately for us and for the patient, the complicated structure of the brain makes cerebral disease a very different affair from disease in and around the abdominal viscera. He goes on to say that to those who are not acquainted with the literature of the subject there occurs a simple answer to the question, "What can we expect from the surgical treatment of epilepsy?" They may say, "Take the recorded cases, note the results, and make your inferences;" but in the case of the surgical treatment of epilepsy statistics are useless. A few successful cases have been reported and even those with undue haste. Von Bergmann and Sahli have given up the attempt to tabulate the results of operations upon epileptic patients. I started out on the same path a few months ago, but soon found it would be love's labour lost. In this matter of cure of epilepsy after operation, the memory of medical men is not as reliable as it fortunately is with regard to most other diseases. A distinguished surgeon stated recently before one association that he knew of a case of traumatic epilepsy in which the cure was of ten years' duration, and before another society, a few days later, that the longest cure that he knew of was of three years' duration. But what of the innumerable failures? And if the attacks have been inhibited for a number of weeks or months, is even this temporary success to be put to the credit of the operation? The author does well to call to mind the fact that epilepsy is a symptom not a disease, and that it is often merely one of a number of symptoms pointing to organic disease of the brain, to tumour, hæmorrhage, abscess, or widespread meningitis and sclerosis. But though it is impossible to say that where there is a local pathological centre there are not also secondary changes in the brain tissues generally, which may at any time years after show themselves by the recurrence of fits, yet it is the method of excision of the initial focal lesion which is recommended in Jacksonian "Epilepsy." If the diseased centre is the only irritable area, the result will probably be a good one; but we have no means of predicting whether or not this will be the case. And to make matters worse, excision of a centre means loss of function. You may not cure the epilepsy, but you are very apt to paralyse the convulsed parts; but this function of the excised part is very apt to be assumed by other parts of the brain, particularly in young persons, and among older persons the patient if left to make his choice will prefer local paralysis to a severe epilepsy. The practical conclusions the writer draws from the foregoing are these: 1. In a given case of traumatic or organic lesions, operate as early as possible to prevent the development of secondary sclerosis. 2. If you have not operated at the outset, the onset of epilepsy is a warning that secondary sclerosis has been established; by operation at this time you may avoid an increase of the trouble. 3. Excision of the diseased area is the only rational operation; if all other centres are not in an irritable condition, the operation may be thoroughly successful.

Professor Sachs, however, is a strong advocate of tre-

* New York *Med. Journ.*, Feb. 20, 1892.

phining whenever there is the slightest suspicion that traumatism may have produced depression of bone. As trepanation is not a very dangerous operation, it would be better to do this than to have the slightest doubt. It is better too to prevent epilepsy than to cure it. He allows that after epilepsy, following traumatic injury of the skull or of the brain, has been developed, there is still hope that the epilepsy may, in a few instances, be inhibited by surgical methods.

Certainly many other surgeons who have had practical experience in trephining for epilepsy take a more favourable view of the prospects of the procedure. Thus three cases at St. George's Hospital, under Mr. Pick, have been followed by apparent cure, and only time can show whether the marked temporary benefit will be maintained. One case was that of a dairyman, age 33, who had had a fall from a swing twenty-four years previously. In five years no result followed, but he then began to have fits of an epileptiform character. They increased in frequency. For three months before admission he had had no fit, but he had been taking regularly for three months a mixture of bromide and iodide of potassium. Latterly his health had been bad, he was very depressed, the convulsions appear to have been universal. On the right side there was a scar behind the coronal suture, and after an exploratory incision over the scar a triangular depression was found in his skull, this was trephined. The operation was followed by great improvement, the fits occurred but rarely, although he was observed for three years, and his general condition of health was much improved.

Pedrazzi, of Naples, Arbuthnot Lane, Maglioni, Cannes, report cases followed by good results, but Alexander Miles and Knapp have had no good results in their cases. In one of Dr. Miles' cases a distinct pathological lesion was found, and, so far as could be ascertained, removed. Yet no marked improvement took place in the condition of the patient. He remarks that, of course, it is possible that the pressure of the cyst, which was found, had damaged the cortical centres over which it was placed beyond the point from which they could recover.

